# Role of anthropometric indices as a screening tool for predicting metabolic syndrome among apparently healthy individuals of Karachi, Pakistan

**DOI:** 10.3389/fendo.2023.1223424

**Published:** 2023-10-09

**Authors:** Syed Omair Adil, Kamarul Imran Musa, Fareed Uddin, Kashif Shafique, Asima Khan, Md Asiful Islam

**Affiliations:** ^1^ Department of Community Medicine, School of Medical Sciences, Universiti Sains Malaysia, Kubang Kerian, Kelantan, Malaysia; ^2^ School of Public Health, Dow University of Health Sciences (DUHS), Karachi, Pakistan; ^3^ National Institute of Diabetes & Endocrinology, DUHS, Karachi, Pakistan; ^4^ Public Health Department, Baqai Institute of Diabetology & Endocrinology, Karachi, Pakistan; ^5^ WHO Collaborating Centre for Global Women’s Health, Institute of Metabolism and Systems Research, College of Medical and Dental Sciences, University of Birmingham, Birmingham, United Kingdom

**Keywords:** metabolic syndrome, anthropometry, predictive value of tests, mass screening, Pakistan anthropometric indices, Pakistan

## Abstract

**Introduction:**

Anthropometric indices are affordable and non-invasive methods for screening metabolic syndrome (MetS). However, determining the most effective index for screening can be challenging.

**Objective:**

To investigate the accuracy of anthropometric indices as a screening tool for predicting MetS among apparently healthy individuals in Karachi, Pakistan.

**Methods:**

A community-based cross-sectional survey was conducted in Karachi, Pakistan, from February 2022 to August 2022. A total of 1,065 apparently healthy individuals aged 25 years and above were included. MetS was diagnosed using International Diabetes Federation guidelines. Anthropometric indices were defined based on body mass index (BMI), neck circumference (NC), mid-upper arm circumference (MUAC), waist circumference (WC), waist to height ratio (WHtR), conicity index, reciprocal ponderal index (RPI), body shape index (BSI), and visceral adiposity index (VAI). The analysis involved the utilization of Pearson’s correlation test and independent t-test to examine inferential statistics. The receiver operating characteristic (ROC) analysis was also applied to evaluate the predictive capacities of various anthropometric indices regarding metabolic risk factors. Moreover, the area under the curve (AUC) was computed, and the chosen anthropometric indices’ optimal cutoff values were determined.

**Results:**

All anthropometric indices, except for RPI in males and BSI in females, were significantly higher in MetS than those without MetS. VAI [AUC 0.820 (95% CI 0.78–0.86)], WC [AUC 0.751 (95% CI 0.72–0.79)], WHtR [AUC 0.732 (95% CI 0.69–0.77)], and BMI [AUC 0.708 (95% CI 0.66–0.75)] had significantly higher AUC for predicting MetS in males, whereas VAI [AUC 0.693 (95% CI 0.64–0.75)], WHtR [AUC 0.649 (95% CI 0.59–0.70)], WC [AUC 0.646 (95% CI 0.59–0.61)], BMI [AUC 0.641 (95% CI 0.59–0.69)], and MUAC [AUC 0.626 (95% CI 0.57–0.68)] had significantly higher AUC for predicting MetS in females. The AUC of NC for males was 0.656 (95% CI 0.61–0.70), while that for females was 0.580 (95% CI 0.52–0.64). The optimal cutoff points for all anthropometric indices exhibited a high degree of sensitivity and specificity in predicting the onset of MetS.

**Conclusion:**

BMI, WC, WHtR, and VAI were the most important anthropometric predictors for MetS in apparently healthy individuals of Pakistan, while BSI was found to be the weakest indicator.

## 1 Introduction

Metabolic syndrome (MetS), also known as cardiometabolic syndrome, syndrome X, or insulin resistance syndrome, is one of the challenging public health issues being studied for the last two decades (1, 2). The identification of the factors contributing to the MetS is important as early identification can combat the occurrence of type 2 diabetes and cardiovascular diseases (3).

The term MetS was first described by Reaven in the late 1980s. Reaven introduced the concept of “Syndrome X” and defined it as a cluster of disturbances in glucose and insulin metabolism, dyslipidemia, and hypertension (HTN) (4). After this conception, insulin resistance was considered as the fundamental disorder associated with a set of metabolic abnormalities which not only increased the risk of type 2 diabetes but also contributed to the development of cardiovascular disease before the appearance of hyperglycemia. Thus, hypertriglyceridemia, low high-density lipoprotein (HDL) cholesterol level, increased glycemia, and elevated blood pressure were noted as the center of a cluster of metabolic abnormalities, which resulted due to the insulin resistance (4).

To date, different criteria have been proposed for the screening of MetS. Even though these screening criteria have been proposed by various renowned organizations and researchers and most of them are made up of similar concepts, the predictive role of each criterion remains controversial over time. However, still, the most common individual predicting factors remain the same as revealed by Reaven and other previous researchers. These factors are dyslipidemia (triglycerides and cholesterol), HTN, glucose intolerance, and adiposity. In addition to this, several other factors were added later for the prediction of MetS that includes health behavior like smoking, inflammatory profile, gender, and ethnicity (5–8).

Thus, insulin resistance was the first hallmark in the screening of the MetS (9). Abdominal obesity is later considered as the second most key criteria in initiating the underlying factors associated with the risk of MetS (10). According to this concept, it is hypothesized that abdominal obesity exacerbates insulin resistance and increases the likelihood of the occurrence of the associated diseases (10–13). Due to the inclusion of abdominal obesity as one of the key factors in determination of the risk of MetS, anthropometric indices have also gained popularity while predicting the risk of MetS (14–16). However, different experts have proposed different cutoff points for individual anthropometric measures, which has also created controversy in the predictive role of anthropometric indices (17–19). Factors such as genetic variations, environmental conditions, and socioeconomic diversity played a vital role and should be dealt with consciously while determining the cutoff level of anthropometric indices for the screening of MetS (8). Thus, the cutoff level of anthropometric factors varies as per the population. Therefore, epidemiological studies that assess the role of anthropometric indices for predicting metabolic risks in particular populations are much needed in this situation. The current study was carried out in a metropolitan city of Pakistan to investigate the role of anthropometric indices as a screening tool for predicting MetS among apparently healthy individuals.

## 2 Materials and methods

### 2.1 Study setting and duration

A community-based cross-sectional survey was conducted at different areas of Karachi, Pakistan, from February 2022 to August 2022. Karachi is the capital of the Pakistan province Sindh situated on the Arabian Sea. Karachi serves as a transport hub and commercial and industrial center and is home to Pakistan’s two largest seaports, as well as the busiest airport in Pakistan. It is one of the world’s fastest-growing cities and has communities representing almost every ethnic group in Pakistan.

Multiple screening camps were organized at different locations and places of Karachi to cover all the diverse population and wide areas of Karachi city. Approval from the ethical committee was obtained prior to conducting the study. Moreover, informed consent was obtained from all eligible study participants after explanation of the purpose of the study.

### 2.2 Sample selection

All apparently healthy individuals aged 25 years and above of any gender presenting with no history of diabetes, HTN, malignancy, stroke, cardiovascular diseases, and renal disorders were included, while pregnant women and lactating women were excluded.

Apparently, healthy individuals were defined as those not taking any medication regularly, had no physical disability, and perceived themselves as healthy due to absence of any disease, signs, or symptoms.

### 2.3 Metabolic syndrome

For laboratory investigation, two phlebotomists were arranged in each screening camp to collect the blood samples of those healthy individuals who visited the screening camp with at least 10 h of fasting while a free coupon of Dow University laboratory was distributed to those healthy individuals who came without fasting, for the test on a later day when they are fasting.

MetS was diagnosed using guidelines given by International Diabetes Federation (IDF) (20). The presence of three of the following five characteristics was labeled as positive for MetS. This includes elevated waist circumference (≥90 cm in men and ≥80 cm in women), elevated blood pressure (≥130/85 mmHg or taking prescribed antihypertensive medications), reduced HDL-cholesterol (<1.03 mmol/L in men or <1.29 mmol/L in women, or on lipid lowering medications), elevated triglycerides (≥1.7 mmol mg/dL or on drug treatment), or elevated fasting blood glucose (FBG) (≥100 mg/dL or on glucose lowering medications). Waist circumference (WC) was noted according to the ethnicity specific criteria for Asians.

### 2.4 Anthropometric indices

Anthropometric indices were defined based on body mass index (BMI), neck circumference (NC), mid-upper arm circumference (MUAC), waist circumference (WC), waist to height ratio (WHtR), conicity index (CI), reciprocal ponderal index (RPI), body shape index (BSI), and visceral adiposity index (VAI). WC was measured at the midpoint between the lower rib margin (12th rib) and the iliac crest. Neck circumference (NC) was measured below the laryngeal prominence and perpendicular to the long axis of the neck, and the minimal circumference was recorded to the nearest 0.1 cm. Measurements were performed using an electronic scale (Seca Limited) and a stadiometer (Seca Limited). BMI was calculated as weight divided by the square of height, in kilograms per square meters (kg/m^2^). WHtR was calculated as WC in cm divided by height in cm. CI was calculated as waist/[0.09 × square root of (weight/height)]. RPI was calculated as height divided by cube root of body weight, i.e., height/weight^1/3^. BSI was calculated as WC in meters divided by BMI^2/3^ × square root of height in meters. VAI was calculated using formula: VAI_male_ = [WC (cm)/39.68−1.88BMI (kg/m^2^)] [TG (mmol/L)/1.03][1.31/HDL (mmol/L)]. VAI_female_ = [WC (cm)/36.58−1.89BMI (kg/m^2^)] [TG (mmol/L)/0.81][1.52/HDL (mmol/L)].

### 2.5 Blood sample collection and detection

The blood samples were obtained from eligible study participants following a minimum 8-h overnight fasting. A sterile vacutainer device was used to collect around 10 mL of venous blood. For the FBS test, blood samples were collected in a tube with sodium citrate anticoagulant whereas for the lipid profile, blood was collected in tube without anticoagulant. The samples for FBS were gently mixed with anticoagulant before the serum was separated, all the samples were subjected to centrifugation at 1,500 rpm for 10 min. After analysis, the collected serum was divided into aliquots and kept at −80°C. Metabolic parameters, including blood glucose and lipid profile, were assessed using established biochemical assays following the manufacturer’s instructions. Quality control measures were implemented to ensure accuracy.

### 2.6 Blood pressure measurement

Blood pressure measurements were obtained using digital sphygmomanometers. Participants were seated comfortably with their arm supported at the heart level. Systolic blood pressure (SBP) and diastolic blood pressure (DBP) were recorded as the first Korotkoff sound and the disappearance of sound, respectively.

### 2.7 Data analysis plan

Data was entered, cleaned, and analyzed by using SPSS version 26. Cleaning and coding of the data were done prior to the analysis. Individuals having any missing component of MetS, i.e., blood pressure, fasting plasma glucose, triglyceride, HDL-C, and WC, were excluded. Mean and the standard deviation were reported for anthropometric measurements, and fasting plasma glucose, SBP, DBP, and TG, and HDL-c levels were assessed prior to the conducting of the analysis. The relationship of anthropometric indices with the components of MetS such as SBP, DBP, FBG, TG, and HDL-c was explored using Pearson’s correlation test. The mean difference of anthropometric indices with MetS and its components was explored using independent sample t-test whereas Pearson’s correlation test was applied to see the relationship between anthropometric indices and the quantitative components of MetS. Furthermore, receiver operating characteristic (ROC) analysis was applied to assess the abilities of different anthropometric indices to predict metabolic risk factors. Moreover, optimal cutoff values of the selected anthropometric indices were determined.

## 3 Results

### 3.1 Baseline characteristics of the patients

A total of 1,065 healthy individuals participated in the study. The mean age of the participants was 42.66 ± 12.18 years. There were 667 (62.6%) males and 398 (37.4%) females. The prevalence of elevated waist circumference was found to be 73.9% (95% CI: 71.1−76.5), high FBG level 19.5% (95% CI 17.1−22.0), high TG level 19.6% (95% C.I 17.2−22.1), low HDL-c level 49.9% (95% C.I 46.8−52.9), and high blood pressure 51.1% (95% C.I 48.0−54.1).The prevalence of MetS based on IDF definition was found to be 32.2% (95% C.I 29−35). In males, the prevalence of MetS was found to be 29.8% (95% C.I26.4−33.5), whereas in females, the prevalence of MetS was 36.2% (95% C.I 31.4−41.1). In males, all anthropometric indices (except RPI) were significantly higher in individuals with MetS as compared to non-MetS individuals, whereas in females, all anthropometric indices (except BSI) were significantly higher in individuals with MetS as compared to non-MetS individuals (**Tables 1**, **2**).

**Table 1:** Mean difference of anthropometric indices with metabolic syndrome and its components in males (n = 667).

	BMI, kg/m^2^	NC, cm	MUAC, cm	WC, cm	WHtR	CI	RPI	BSI	VAI
	Mean ± SD	Mean ± SD	Mean ± SD	Mean ± SD	Mean ± SD	Mean ± SD	Mean ± SD	Mean ± SD	Mean ± SD
HTN									
Yes	26.69 ± 4.37	38.26 ± 3.15	30.99 ± 3.84	95.20 ± 11.42	0.56 ± 0.07	1.31 ± 0.11	40.64 ± 2.29	0.08 ± 0.01	2.28 ± 1.31
No	25.49 ± 4.12	37.31 ± 3.87	30.43 ± 3.79	91.98 ± 10.89	0.54 ± 0.07	1.29 ± 0.11	41.24 ± 2.33	0.08 ± 0.01	2.20 ± 1.28
*p-value*	<0.001	0.001	0.061	<0.001	<0.001	0.081	0.001	0.602	0.418
Elevated waist circumference, cm									
Yes	26.12 ± 4.41	37.95 ± 3.51	30.78 ± 4.01	94.12 ± 11.62	0.55 ± 0.07	1.30 ± 0.09	40.96 ± 2.35	0.08 ± 0.01	2.40 ± 1.40
No	26.20 ± 3.98	37.47 ± 3.54	30.62 ± 3.27	92.69 ± 10.27	0.55 ± 0.06	1.28 ± 0.14	40.77 ± 2.26	0.08 ± 0.02	1.81 ± 0.75
*p-value*	0.839	0.129	0.626	0.151	0.525	0.177	0.360	0.190	<0.001
Reduced HDL									
Yes	27.51 ± 4.15	37.09 ± 3.49	30.06 ± 4.11	89.44 ± 11.22	0.53 ± 0.06	1.27 ± 0.11	41.42 ± 2.29	0.08 ± 0.01	1.26 ± 0.11
No	25.10 ± 4.11	38.79 ± 3.34	31.64 ± 3.21	99.42 ± 8.55	0.59 ± 0.05	1.34 ± 0.09	40.25 ± 2.21	0.08 ± 0.00	1.34 ± 0.09
*p-value*	<0.001	<0.001	<0.001	<0.001	<0.001	<0.001	<0.001	<0.001	<0.001
Increased FBP									
Yes	27.63 ± 4.21	39.01 ± 3.39	31.73 ± 3.77	98.33 ± 12.32	0.57 ± 0.07	1.32 ± 0.11	40.24 ± 2.15	0.08 ± 0.01	2.51 ± 1.53
No	25.80 ± 4.25	37.55 ± 3.49	30.52 ± 3.81	92.69 ± 10.78	0.55 ± 0.06	1.29 ± 0.10	41.07 ± 2.35	0.08 ± 0.00	2.18 ± 1.23
*p-value*	<0.001	<0.001	<0.001	<0.001	<0.001	0.017	<0.001	0.387	0.012
Increased TG									
Yes	27.09 ± 4.21	38.41 ± 3.30	31.30 ± 3.75	96.68 ± 10.69	0.57 ± 0.06	1.31 ± 0.09	40.45 ± 2.18	0.08 ± 0.01	3.90 ± 1.62
No	25.83 ± 4.28	37.63 ± 3.57	30.56 ± 3.84	92.79 ± 11.33	0.55 ± 0.07	1.29 ± 0.11	41.07 ± 2.36	0.08 ± 0.02	1.71 ± 0.43
*p-value*	0.001	0.015	0.032	<0.001	0.001	0.047	0.003	0.381	<0.001
Metabolic syndrome									
Yes	28.19 ± 3.95	39.01 ± 3.23	32.25 ± 3.68	100.47 ± 9.08	0.59 ± 0.05	1.33 ± 0.09	39.92 ± 2.03	0.08 ± 0.00	3.26 ± 1.72
No	25.28 ± 4.15	37.32 ± 3.52	30.10 ± 3.71	90.89 ± 10.93	0.54 ± 0.06	1.28 ± 0.11	41.34 ± 2.32	0.08 ± 0.01	1.81 ± 0.72
*p-value*	<0.001	<0.001	<0.001	<0.001	<0.001	<0.001	<0.001	0.002	<0.001

BMI, body mass index; BP, blood pressure; BSI, body shape index; CI, conicity index; HDL-c, high-density lipoprotein cholesterol; MUAC, mid upper arm circumference; NC, neck circumference; RPI, reciprocal ponderal index; TG, triglycerides; VAI, visceral adiposity index, WC, waist circumference; WHtR, waist to height ratio.

**Table 2:** Mean difference of anthropometric indices with metabolic syndrome and its components in females (n = 398).

	BMI, kg/m^2^	NC, cm	MUAC, cm	WC, cm	WHtR	CI	RPI	BSI	VAI
	Mean ± SD	Mean ± SD	Mean ± SD	Mean ± SD	Mean ± SD	Mean ± SD	Mean ± SD	Mean ± SD	Mean ± SD
HTN									
Yes	28.88 ± 4.97	36.10 ± 3.80	31.51 ± 4.25	93.41 ± 12.65	0.61 ± 0.08	1.29 ± 0.15	38.42 ± 2.50	0.08 ± 0.01	2.54 ± 0.99
No	27.01 ± 4.69	35.23 ± 4.09	30.19 ± 4.07	88.20 ± 12.30	0.57 ± 0.09	1.25 ± 0.13	39.47 ± 2.52	0.07 ± 0.01	2.44 ± 1.07
*p-value*	<0.001	0.029	0.002	<0.001	<0.001	0.003	<0.001	0.060	0.335
Elevated waist circumference, cm									
Yes	28.20 ± 4.73	35.90 ± 4.04	31.23 ± 4.13	92.47 ± 11.34	0.59 ± 0.07	1.28 ± 0.12	38.82 ± 2.40	0.08 ± 0.01	2.58 ± 1.06
No	26.84 ± 5.27	34.83 ± 3.74	29.51 ± 4.14	85.08 ± 14.69	0.55 ± 0.10	1.21 ± 0.18	39.51 ± 2.92	0.07 ± 0.01	2.21 ± 0.91
*p-value*	0.015	0.019	<0.001	<0.001	<0.001	<0.001	0.018	<0.001	0.001
Reduced HDL									
Yes	26.27 ± 4.79	34.86 ± 4.19	29.77 ± 4.46	83.67 ± 11.81	0.54 ± 0.08	1.21 ± 0.13	39.81 ± 2.64	0.07 ± 0.01	2.82 ± 1.23
No	28.82 ± 4.74	36.09 ± 3.79	31.41 ± 3.90	94.79 ± 11.33	0.61 ± 0.07	1.31 ± 0.12	38.50 ± 2.38	0.08 ± 0.00	2.27 ± 0.83
*p-value*	<0.001	0.003	<0.001	<0.001	<0.001	<0.001	<0.001	<0.001	<0.001
Increased FBP									
Yes	29.28 ± 5.01	36.17 ± 4.06	31.68 ± 4.28	93.29 ± 13.95	0.60 ± 0.10	1.27 ± 0.13	38.35 ± 2.50	0.08 ± 0.01	2.81 ± 1.19
No	27.47 ± 4.83	35.48 ± 3.95	30.54 ± 4.15	89.82 ± 12.29	0.58 ± 0.08	1.26 ± 0.14	39.17 ± 2.56	0.07 ± 0.01	2.40 ± 0.97
*p-value*	0.003	0.161	0.028	0.027	0.048	0.914	0.013	0.433	0.001
Elevated TG									
Yes	29.28 ± 4.98	36.08 ± 3.83	32.60 ± 4.36	94.50 ± 11.25	0.60 ± 0.07	1.27 ± 0.10	38.19 ± 2.40	0.08 ± 0.01	4.44 ± 1.33
No	27.59 ± 4.84	35.56 ± 4.01	30.54 ± 4.12	90.04 ± 12.82	0.58 ± 0.09	1.26 ± 0.15	39.11 ± 2.57	0.07 ± 0.01	2.23 ± 0.66
*p-value*	0.004	0.407	0.002	0.026	0.099	0.837	0.025	0.559	<0.001
Metabolic syndrome									
Yes	29.26 ± 4.39	36.40 ± 3.98	31.92 ± 3.98	94.55 ± 9.08	0.61 ± 0.07	1.29 ± 0.11	38.29 ± 2.19	0.08 ± 0.00	2.97 ± 1.22
No	27.04 ± 5.01	35.18 ± 3.93	30.14 ± 4.18	88.27 ± 13.88	0.56 ± 0.09	1.25 ± 0.15	39.45 ± 2.65	0.07 ± 0.01	2.21 ± 0.78
*p-value*	<0.001	0.003	<0.001	<0.001	<0.001	0.002	<0.001	0.073	<0.001

BMI, body mass index; BP, blood pressure; BSI, body shape index; CI, conicity index; HDL-c, high-density lipoprotein cholesterol; HTN, hypertension; MUAC, mid upper arm circumference; NC, neck circumference; RPI, reciprocal ponderal index; TG, triglycerides; VAI, visceral adiposity index; WC, waist circumference; WHtR, waist to height ratio.

### 3.2 Correlation analysis of anthropometric indices with quantitative components of MetS

In males, most of the anthropometric indices except BSI were significantly correlated with different components of MetS. A strong negative correlation of HDL-c was observed with BMI (r = −0.960, p-value 0.013) and NC (r = -0.820, p-value 0.034) (**Table 3**).

**Table 3:** Correlation analysis of anthropometric indices with quantitative components of metabolic syndrome (n = 1,065).

	BMI, kg/m^2^	NC, cm	MUAC, cm	WC, cm	WHtR	CI	RPI	BSI	VAI
Males									
SBP	0.213**	0.181**	0.170**	0.246**	0.255**	0.151**	-0.214**	0.075	0.026
DBP	0.203**	0.106*	0.169**	0.180**	0.183**	0.061	-0.203**	-0.008	0.057
FPG	0.201**	0.186**	0.177**	0.204**	0.183**	0.082*	-0.177**	0.013	0.083*
HDL-c	−0.960*	−0.820*	−0.102*	−0.062	−0.048	0.017	0.081*	0.045	−0.470**
TG	0.171**	0.115*	0.127*	0.174**	0.177**	0.085*	−0.166**	0.027	0.932**
Females									
SBP	0.212**	0.140*	0.165*	0.276**	0.285**	0.207**	−0.215**	0.145*	0.033
DBP	0.133**	0.093	0.172*	0.205**	0.185**	0.156*	−0.122*	0.116*	0.677
FPG	0.163*	0.053	0.075	0.110*	0.097	−0.005	−0.136*	−0.53	0.172*
HDL-c	−0.013	−0.080	−0.005	−0.095	−0.062	−0.084	−0.005	−0.075	−0.584**
TG	0.172*	0.064	0.157*	0.105*	0.094	−0.005	−0.145*	−0.05	0.844**

BMI, body mass index; BP, blood pressure; BSI, body shape index; CI, conicity index; HDL-c, high-density lipoprotein cholesterol; MUAC, mid upper arm circumference; NC, neck circumference; RPI, reciprocal ponderal index; TG, triglycerides; VAI, visceral adiposity index; WC, waist circumference; WHtR, waist to height ratio. *p-value < 0.05, **p-value < 0.001

In females, SBP and DBP were significantly correlated with most of the anthropometric indices. A moderate negative correlation was observed in between HDL-c and VAI (r = −0.584, p-value <0.001), whereas a strong positive significant correlation of TG was observed with VAI in both males (r = 0.932, p-value <0.001) and females (r = 0.844, p-value <0.001) (**Table 3**).

### 3.3 Screening ability of anthropometric indices for metabolic abnormality and its components

**Figure 1:**
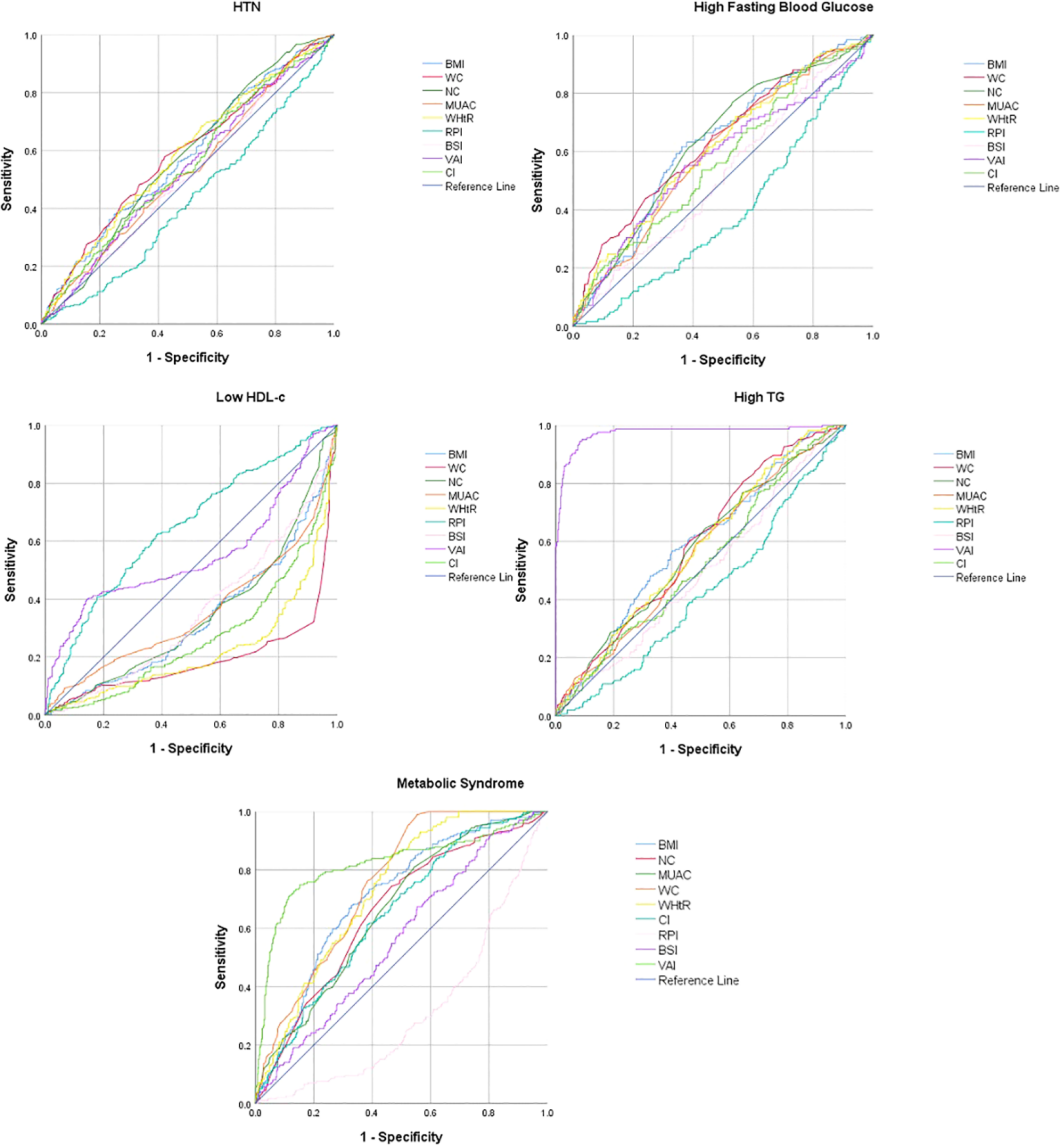
Receiver operating curve for the prediction of metabolic syndrome and its components in males.

In males, most of the anthropometric indices significantly predicted HTN, reduced HDL-c, increased FBG, high TG, and MetS. WC [0.585 (0.54−0.63)], WHtR [0.583 (0.54−0.63)], BMI [0.577 (0.53−0.62)], NC [0.564 (0.51−0.62)], and CI [0.547 (0.50−0.59)] had significantly higher AUC to predict HTN in males. RPI and CI had the significantly higher AUC to predict reduced HDL-c, i.e., 0.648 (0.61−0.69) and 0.552 (0.51−0.59), respectively. WC [0.636 (0.58−0.69)], NC [0.631 (0.58−0.68)], BMI [0.627 (0.57−0.67)], and WHtR [0.613 (0.56−0.67)] had significantly higher AUC to predict high FBG, while VAI had the significantly higher AUC to predict high TG in males, i.e., 0.974 (0.96−0.99) (**Table 4**; **Figure 1**).

**Table 4:** Area under the receiver operating curve for anthropometric indices with metabolic syndrome and its components (n = 1,065).

	HTN	Reduced HDL-c	High FBG	High TG	Metabolic syndrome
	AUC (95% CI)	AUC (95% CI)	AUC (95% CI)	AUC (95% CI)	AUC (95% CI)
Males					
BMI, kg/m^2^	0.577 (0.53–0.62)*	0.331 (0.29–0.37)**	0.627 (0.57–0.67)**	0.586 (0.54–0.64)*	0.708 (0.66–0.75)**
NC, cm	0.564 (0.51–0.62)*	0.344 (0.31–0.39)**	0.631 (0.58–0.68)**	0.569 (0.52–0.62)*	0.656 (0.61–0.70)**
MUAC, cm	0.531 (0.49–0.58)	0.354 (0.31–0.39)**	0.599 (0.55–0.65)*	0.555 (0.51–0.61)*	0.661 (0.62–0.70)**
WC, cm	0.585 (0.54–0.63)**	0.194 (0.16–0.23)**	0.636 (0.58–0.69)**	0.588 (0.54–0.64)*	0.751 (0.72–0.79)**
WHtR	0.583 (0.54–0.63)**	0.225 (0.19–0.26)**	0.613 (0.56–0.67)**	0.576 (0.53–0.62)*	0.732 (0.69–0.77)**
CI	0.547 (0.50–0.59)*	0.552 (0.51–0.59)*	0.568 (0.51–0.62)*	0.526 (0.48–0.57)	0.648 (0.61–0.69)**
RPI	0.427 (0.38–0.47)*	0.648 (0.61–0.69)**	0.394 (0.34–0.45)**	0.423 (0.37–0.47)*	0.313 (0.27–0.36)**
BSI	0.512 (0.47–0.56)	0.352 (0.31–0.39)**	0.519 (0.46–0.57)	0.493 (0.44–0.54)	0.563 (0.52–0.61)*
VAI	0.533 (0.49–0.58)	0.552 (0.51–0.59)*	0.574 (0.52–0.63)*	0.974 (0.96–0.99)**	0.820 (0.78–0.86)**
Females					
BMI, kg/m^2^	0.612 (0.56–0.69)**	0.34 (0.29–0.40)**	0.606 (0.54–0.67)*	0.626 (0.54–0.71)*	0.641 (0.59–0.69)**
NC, cm	0.572 (0.53–0.62)*	0.401 (0.34–0.46)*	0.549 (0.48–0.62)	0.544 (0.46–0.63)	0.580 (0.52–0.64)*
MUAC, cm	0.593 (0.54–0.65)*	0.387 (0.33–0.44)**	0.576 (0.51–0.65)*	0.635 (0.55–0.72)*	0.626 (0.57–0.68)**
WC, cm	0.620 (0.56–0.67)**	0.240 (0.19–0.29)**	0.563 (0.48–0.64)	0.620 (0.54–0.70)*	0.646 (0.59–0.61)**
WHtR	0.636 (0.58–0.69)**	0.253 (0.20–0.30)**	0.551 (0.47–0.63)	0.575 (0.49–0.66)	0.649 (0.59–0.70)**
CI	0.588 (0.53–0.64)*	0.584 (0.53–0.64)*	0.485 (0.41–0.56)	0.502 (0.42–0.58)	0.585 (0.53–0.64)*
RPI	0.381 (0.33–0.44)**	0.651 (0.59–0.71)**	0.410 (0.34–0.48)*	0.404 (0.32–0.49)*	0.363 (0.31–0.42)**
BSI	0.555 (0.49–0.61)	0.344 (0.29–0.39)**	0.458 (0.39–0.53)	0.474 (0.39–0.55)	0.547 (0.49–0.60)
VAI	0.559 (0.50–0.62)*	0.584 (0.53–0.64)*	0.597 (0.53–0.67)*	0.962 (0.93–0.99)**	0.693 (0.64–0.75)**

BMI, body mass index; BP, blood pressure; BSI, body shape index; CI, conicity index; FBG, fasting blood glucose; HDL-c, high-density lipoprotein cholesterol; HTN, hypertension; MUAC, mid upper arm circumference; NC, neck circumference; RPI, reciprocal ponderal index; TG, triglycerides; VAI, visceral adiposity index; WC, waist circumference; WHtR: waist to height ratio. *p-value <0.05, **p-value <0.001.

**Figure 2:**
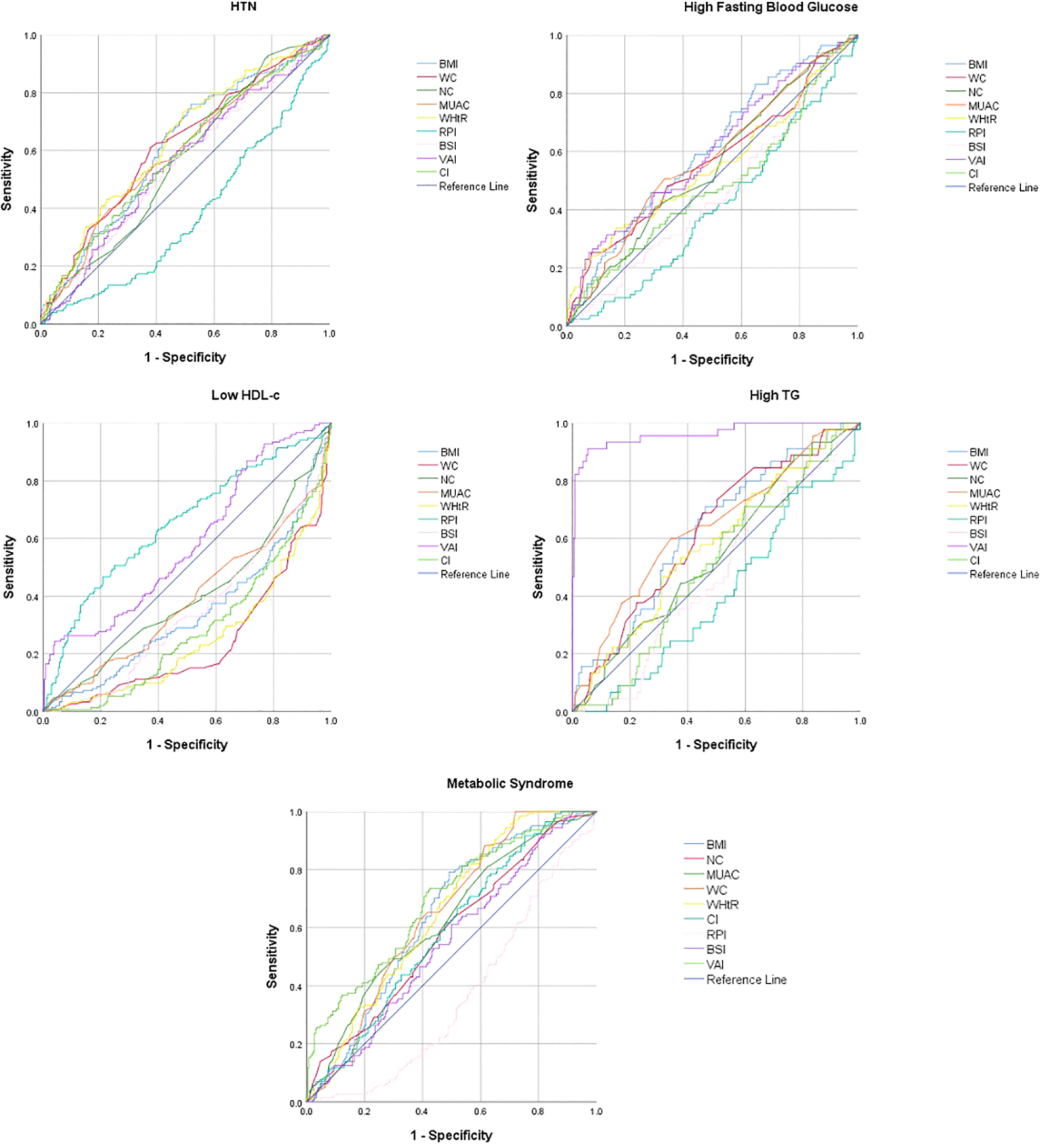
Receiver operating curve for the prediction of metabolic syndrome and its components in females.

In females, most of the anthropometric indices significantly predicted reduced HDL-c whereas except BSI most of the other anthropometric indices significantly predicted HTN and MetS. Only a few anthropometric indices significantly predicted high FBG and high TG. WHtR [0.636 (0.58−0.69)], WC [0.620 (0.56−0.67)], and BMI [0.612 (0.56−0.69)] had the significantly higher AUC to predict HTN in females. RPI [0.651 (0.59−0.71)] had the significantly higher AUC to predict reduced HDL-c in females. BMI [0.606 (0.54−0.67)] had the significantly higher AUC to predict high FBG in females, while VAI [0.962 (0.93−0.99)] had the significantly higher AUC to predict high TG in females (**Table 4**; **Figure 2**).

VAI [0.820 (0.78−0.86)], WC [0.751 (0.72−0.79)], WHtR [0.732 (0.69−0.77)], and BMI [0.708 (0.66−0.75)] had significantly higher AUC for predicting MetS in males, whereas VAI [0.693 (0.64−0.75)], WHtR [0.649 (0.59−0.70)], WC [0.646 (0.59−0.61)], BMI [0.641 (0.59−0.69)], and MUAC [0.626 (0.57−0.68)] had significantly higher AUC for predicting MetS in females (**Table 4**; **Figures 1**, **2**).

### 3.4 Optimal cutoff value of anthropometric indicators for the identification of MetS

The optimal cutoff value of each anthropometric indices for screening of MetS for BMI was 25.02 in men and 25.00 in women, for NC 36.15 in men and 33.01 in women, for MUAC 27.97 in men and 26.15 in women, for WC 90.08 in men and 85.5 in women, for WHtR 0.05 in men and 0.05 in women, for CI 1.21 in men and 1.18 in women, for RPI 39 in men and 37.01 in women, for BSI 0.08 in men and 0.07 in women, and for VAI was 1.7 in men and 2.00 in women (**Table 5**).

**Table 5:** Optimal cutoff values and sensitivity and specificity for the prediction of metabolic syndrome.

	Cutoff point	Sensitivity	1-Specificity
Males			
BMI, kg/m^2^	25.02	0.76	0.46
NC, cm	36.15	0.84	0.61
MUAC, cm	27.97	0.92	0.73
WC, cm	90.08	0.96	0.53
WHtR	0.50	0.99	0.70
CI	1.21	0.94	0.79
RPI	39.00	0.67	0.84
BSI	0.08	0.69	0.58
VAI	1.70	0.87	0.61
Females			
BMI, kg/m^2^	25.00	0.83	0.57
NC, cm	33.01	0.75	0.65
MUAC, cm	26.15	0.92	0.82
WC, cm	85.5	0.88	0.65
WHtR	0.50	0.98	0.76
CI	1.18	0.85	0.72
RPI	37.01	0.71	0.79
BSI	0.07	0.93	0.84
VAI	2.00	0.89	0.68

## 4 Discussion

Though studies have been conducted internationally for a decade to assess the role of anthropometric indices in predicting the risk of development of MetS. However, the screening criteria and optimal cutoff level are still controversial and debatable (4). The major reason behind this discrepancy is the involvement of too many factors with diseases associated with the MetS. Moreover, the geographical variation and evolutions in the factors associated with the MetS also cause trouble in determination of the effective criteria for the screening of the disease (1–4). In addition, there is scarcity of studies from Pakistan that has reported the role of anthropometric indices in predicting the risk of development of MetS in Pakistani population. This community-based cross-sectional study was thus designed to predict the role of anthropometric indices in screening of MetS in Pakistani population.

The findings of the study have revealed that the mean values of all anthropometric indices (except BSI in females) were significantly higher in individuals with MetS in both men and women. These findings are consistent with previous studies that have also reported higher mean values of anthropometric indices in individuals with MetS (16, 21, 22).

As per the current study findings, BMI, NC, and VAI were the three anthropometric indices with significantly higher mean values in male individuals with presence of four components of MetS, followed by WC, WHtR, and RPI with significantly higher mean values in three components whereas MUAC and CI had significantly higher mean values in two components of MetS. Furthermore, BMI, MUAC, WC, and RPI had significantly higher mean values in all five components of MetS in females. WHtR and VAI had significantly higher mean values in four whereas CI and RPI had significantly higher mean values in three components of MetS. BSI was the only anthropometric variable with least mean values in both men and women. Though there are not many studies available on the topic from Pakistan, in a hospital-based study by Hai et al. in Karachi, Pakistan, prevalence of MetS was found to be very high in overweight and obese patients. Moreover, the majority of patients with MetS were found to have higher NC (23).

According to the current study findings, VAI, WC, WHtR, and BMI had significantly higher AUC for predicting MetS in males whereas VAI, WHtR, WC, BMI, and MUAC had significantly higher AUC for predicting MetS in females. Our results are consistent with previously published literature (17, 24, 25). In our study, ABSI was the only indicator that was not found to be a good predictor for MetS. This finding is also supported by previously published findings by various previous studies (17, 26–28).

In the current study, a strong negative correlation of HDL-c was observed with BMI and NC in males whereas a moderate negative correlation was observed in between HDL-c and VAI. In females, SBP and DBP were significantly correlated with most of the anthropometric indices. A strong positive significant correlation of TG was observed with VAI in both genders. Our study findings on correlation of anthropometric indices with MetS are somewhat similar to the findings of previous studies (21, 28–30).

Body rounded index (BRI) was also found to be an important predictor variable as reported in previous studies (28, 30, 31). However, the current study did not report findings of BRI which should be covered in future studies.

The current study also explored the optimal cutoff level for anthropometric indices, which reported that the examined anthropometric indices had optimal cutoff points that displayed a relatively high level of sensitivity and specificity value when predicting the occurrence of MetS. A notable gender-based disparity was identified in the measurements of NC, WC, CI, and VAI, highlighting the necessity of utilizing gender-specific reference values in clinical settings. Somewhat similar findings were also reported in other studies as well (16, 21, 22).

Overall, these anthropometric indices are useful tools for identifying individuals at risk for MetS and other health problems related to obesity. Furthermore, the assessment of anthropometric indices is relatively simple, non-invasive, and inexpensive compared to other measures of body composition such as dual-energy X-ray absorptiometry or magnetic resonance imaging. This makes anthropometric indices a practical tool for identifying individuals at risk for MetS in clinical practice and in population-based studies.

### 4.1 Limitations and strength

The results of the current study might be limited by the use of cross-sectional design, as the temporal association between the studied variables and disease outcome, in addition to the causality, cannot be determined. Also, the ROC curves to figure out the population-specific cutoff values are used in different countries; however, the concern is that these cutoff points might differ based on differences in population characteristics such as the disease prevalence in the studied population, lifestyle preferences, genetic factors, and environmental and sociodemographic factors. Lastly, there are much more men than women in the current study in terms of proportion. Although efforts were made to choose a representative sample, a number of practical considerations, such as participant availability and desire to participate, may have had an impact on the recruitment of study participants. Despite these limitations, as per our understanding, the current study is one of the first studies of its kind that has reported the optimal cutoff level of anthropometric indices and its predictive role in MetS and its components. Moreover, the use of prospective study data is also one of the important strengths of this study in addition to larger sample size which ensures that the information gathered is accurate and only obtained for research. Lastly, as MetS is considerably prevalent in Pakistan as evident by a recent systematic review and meta-analysis (32), advocating the current study findings not only among healthcare providers but in general population too can lead to prevention and early detection of MetS and its components.

## 5 Conclusion

Based on the findings of the current study, BMI, WC, WHtR, and VAI were the most important anthropometric predictors for MetS in apparently healthy individuals of Pakistan, while BSI was found to be the weakest indicator. Both WC and WHtR also had higher sensitivity for MetS screening. The affordable nature of these indices could facilitate improved early detection of MetS, potentially aiding in the prevention of both the MetS itself and its associated complications.

## Data availability statement

The original contributions presented in the study are included in the article/supplementary material. Further inquiries can be directed to the corresponding authors.

## Ethics statement

The studies involving humans were approved by Ethics Committee of Dow University of Health Sciences (IRB-2332/DUHS/Approval/2021/670). The studies were conducted in accordance with the local legislation and institutional requirements. The participants provided their written informed consent to participate in this study.

## Author contributions

Conceptualization: SOA, KM, FU, AK, KS; methodology: SOA, KM, FU, KS, MI; manuscript writing and analysis: SOA; writing—review and editing: KM, KS, FU, AK, MI. All authors have read and agreed to the published version of the manuscript.
